# *sel-11* and *cdc-42*, Two Negative Modulators of LIN-12/Notch Activity in *C. elegans*

**DOI:** 10.1371/journal.pone.0011885

**Published:** 2010-07-29

**Authors:** Min Sung Choi, Andrew S. Yoo, Iva Greenwald

**Affiliations:** 1 Department of Biological Sciences, Howard Hughes Medical Institute, Columbia University College of Physicians and Surgeons, New York, New York, United States of America; 2 Integrated Program in Cellular, Molecular, and Biophysical Studies, Howard Hughes Medical Institute, Columbia University College of Physicians and Surgeons, New York, New York, United States of America; 3 Department of Biochemistry and Molecular Biophysics, Howard Hughes Medical Institute, Columbia University College of Physicians and Surgeons, New York, New York, United States of America; Brown University, United States of America

## Abstract

**Background:**

LIN-12/Notch signaling is important for cell-cell interactions during development, and mutations resulting in constitutive LIN-12/Notch signaling can cause cancer. Loss of negative regulators of *lin-12/Notch* activity has the potential for influencing cell fate decisions during development and the genesis or aggressiveness of cancer.

**Methodology/Principal Findings:**

We describe two negative modulators of *lin-12* activity in *C. elegans*. One gene, *sel-11*, was initially defined as a suppressor of a *lin-12* hypomorphic allele; the other gene, *cdc-42*, is a well-studied Rho GTPase. Here, we show that SEL-11 corresponds to yeast Hrd1p and mammalian Synoviolin. We also show that *cdc-42* has the genetic properties consistent with negative regulation of *lin-12* activity during vulval precursor cell fate specification.

**Conclusions/Significance:**

Our results underscore the multiplicity of negative regulatory mechanisms that impact on *lin-12/Notch* activity and suggest novel mechanisms by which constitutive *lin-12/Notch* activity might be exacerbated in cancer.

## Introduction

LIN-12/Notch signaling plays multiple roles in vulval development in *C. elegans*. Canonical roles for this signaling system in vulval development are the specification of a single anchor cell (AC), which is required for vulval induction, and the specification of the 2° vulval precursor cell (VPC) type. Numerous feedback loops and functional redundancies make vulval development relatively insensitive to small perturbations in signaling pathways. Thus, an individual regulator often does not cause an overt phenotype when it is removed in an otherwise wild-type genetic background, but its function may be revealed when it is removed in a sensitized genetic background. In *C. elegans*, a few core components of LIN-12/Notch signaling have been identified in phenotype-based genetic screens, but most core components and modulators have been identified as suppressors or enhancers in sensitized backgrounds (reviewed in [1]).

Mutations resulting in constitutive or elevated LIN-12/Notch signaling can cause cancer (reviewed in [2], [3]). In principle, abrogation of any system of negative regulation of *lin-12/Notch* activity has the potential for contributing to the development or aggressiveness of cancer. For example, there is some evidence that increased expression of the human SEL-1 ortholog, SEL1L, correlates with a decrease in tumor aggressiveness (reviewed in [4]), but how this correlation relates to effects on Notch activity is not clear. A compelling example is afforded by the ubiquitin ligase SEL-10/Fbw7, which targets LIN-12/Notch directly for proteasome-mediated degradation ([5]; reviewed in [6]). Point mutations in the extracellular domain of NOTCH1 are one cause of T cell acute lymphoblastic leukemia (T-ALL); when patients with these mutations relapse after chemotherapy, they generally have mutations in FBW7. Patients presenting with NOTCH1 mutations that remove the Fbw7 binding site do not have Fbw7 mutations upon relapse. These observations have implicated the loss of the negative regulation of NOTCH1 in patients whose T-ALL tumors are drug-resistant after relapse [7].

Here, we describe two negative modulators of *lin-12* activity. One gene, *sel-11*, was initially defined as a suppressor of a *lin-12* hypomorphic allele [8]. The other, *cdc-42*, is a well-studied Rho GTPase that we find has an effect on *lin-12* activity that appears to be distinct from other roles of *cdc-42* in vulval development described previously [9]. Our results underscore the multiplicity of mechanisms that impact on *lin-12/Notch* activity and suggest novel mechanisms by which aberrations in *lin-12/Notch* activity might be exacerbated in cancer.

## Results

**Figure 1:**
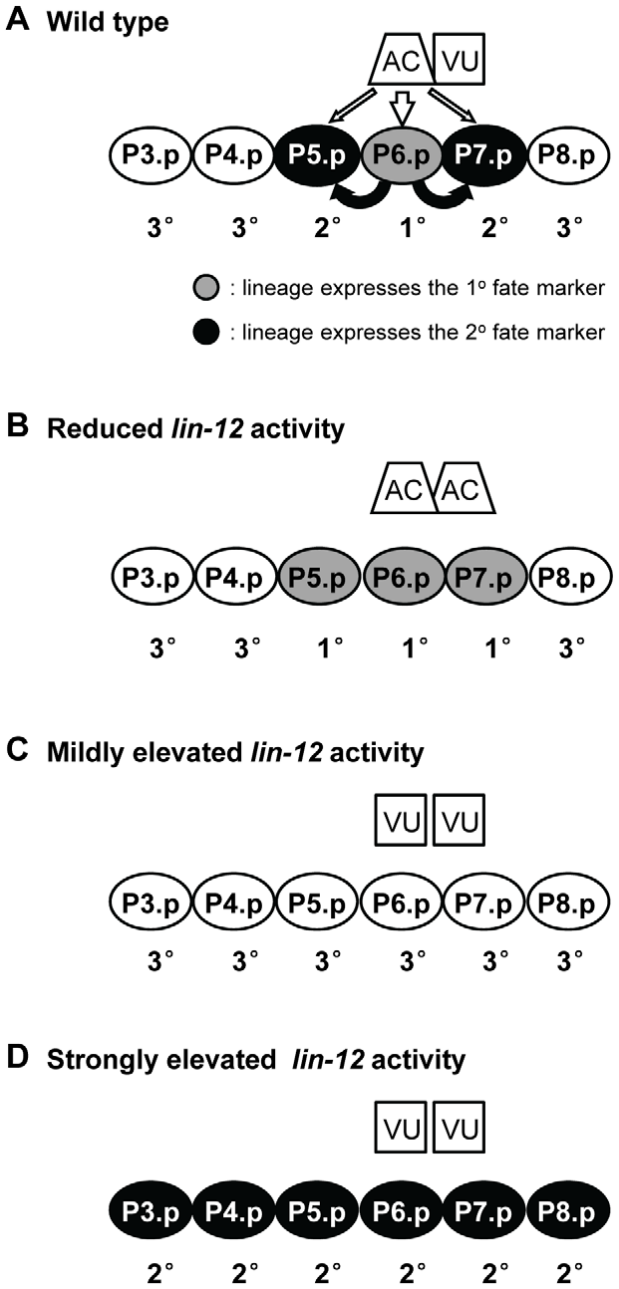
Signaling events that specify Vulval Precursor Cell (VPC) fates, and cell fate changes dependent on *lin-12* activity levels. (A) In wild-type *C. elegans*, vulval development is initiated in the early L3 stage when the anchor cell (AC) signals to the underlying VPCs via an inductive EGF signal, LIN-3 (white arrows). As a result, P6.p adopts the 1° cell fate. P6.p, in turn, sends a lateral signal (black arrows) to its neighboring VPCs, P5.p and P7.p, activating LIN-12 and causing them to adopt the 2° fate. During the AC/VU fate decision in the somatic gonad, the VU fate is determined by high *lin-12* signaling. In wild-type animals, this results in 1 AC. (B) When *lin-12* activity is reduced, a VU is often not specified, resulting in 2 ACs; in addition, as a result of increased inductive signaling from 2 ACs and/or reduced lateral signaling due to lower *lin-12* activity, ectopic VPCs adopt the 1° fate at the expense of the 2° fate. The *lin-12(n676n930)* allele shows both aspects of this phenotype at 25°C [10]. (C) When *lin-12* activity is mildly elevated, the AC fate is lost, and a VU is specified in its place. Since the inductive signal is lost, the underlying VPCs all adopt the 3° fate. *lin-12(n379)* and *lin-12(n676)* are examples of weak hypermorphic alleles, as is the *lin-12(n676n930)* allele at 15°C [10], [18]. (D) However, when *lin-12* activity is strongly elevated, multiple VPCs are induced to adopt the 2° cell fate even though the AC is absent [18].

The genetic analysis of modulators of *lin-12* activity involves combinations with different *lin-12* alleles. Such alleles reduce or elevate *lin-12* activity, and their effects on hallmark cell fate decisions are shown in Fig. 1. We first describe the genetic analysis that establishes *hrd-1* as *sel-11*. We then describe genetic interactions between *cdc-42* and *lin-12*.

### Demonstration that the negative regulator *sel-11* encodes Hrd1p/Synoviolin

Mutations in *sel-11* were originally identified in a genetic screen for negative regulators of *lin-12* activity [8]. This screen was based on suppression of the egg-laying defective (Egl) phenotype caused by the hypomorphic allele, *lin-12(n676n930)* [10]; the properties of this allele are described further below. Extensive genetic analysis of the interactions between *sel-11* and multiple *lin-12* alleles in different cellular contexts established *sel-11* as a negative regulator of *lin-12* activity, but its molecular identity was not determined [8].

We molecularly identified *sel-11* serendipitously because it is adjacent to the microRNA gene *mir-61*. A previous study in our laboratory concluded that *mir-61* is expressed in response to LIN-12 activation and targets VAV-1, a negative regulator of *lin-12* activity [11]. Our view of the role of *mir-61* in vulval development was challenged by Miska et al. [12], who reported that a deletion of *mir-61*, *nDf59*, does not cause overt vulval lineage defects. We independently confirmed that *nDf59* exhibits overtly normal vulval development by examining canonical 1°, 2°, and 3° cell fate markers (Table 1). However, as described below, our further analysis of *nDf59* revealed that it removed *sel-11*, a negative regulator of *lin-12* activity, and it is therefore problematic to use *nDf59* to draw inferences about *mir-61/250* function.

**Table 1:** Vulval cell fate marker expression in *nDf59* is normal compared to control animals.

**1° fate marker (** ***ayIs4 [egl-17::gfp]*** **): scored at Pn.px**				
	% P6.px only	% No expression	% P5.px and P6.px	N
*ayIs4*	91.1	8.9	0	56
*ayIs4; nDf59*	96.2*	1.9	1.9	52
**2° fate marker (** ***nIs106 [lin-11::gfp]*** **): scored at Pn.pxx**				
	% P5.pxx and/or P7.pxx	% P(5,6,7).pxx		N
*nIs106*	100	0		33
*nDf59; nIs106*	95.5	4.5#		44
**3° fate marker (** ***arIs101 [K09H11.1::yfp]*** **): scored at Pn.px-Pn.pxx**				
	% Descendants of P3.p, P4.p, and P8.p			N
*arIs101*	100			53
*nDf59; arIs101*	100&			49

*1 animal had an anteriorly shifted AC above P5.px, and accordingly expression was seen in only P5.px.

# By Fisher's exact test, not significantly different from *nIs106* (*P*>0.5).

& 2/49 animals had an anteriorly shifted AC located above P5.p, and accordingly showed expression in descendants of P3.p, P7.p, and P8.p.

**Figure 2:**
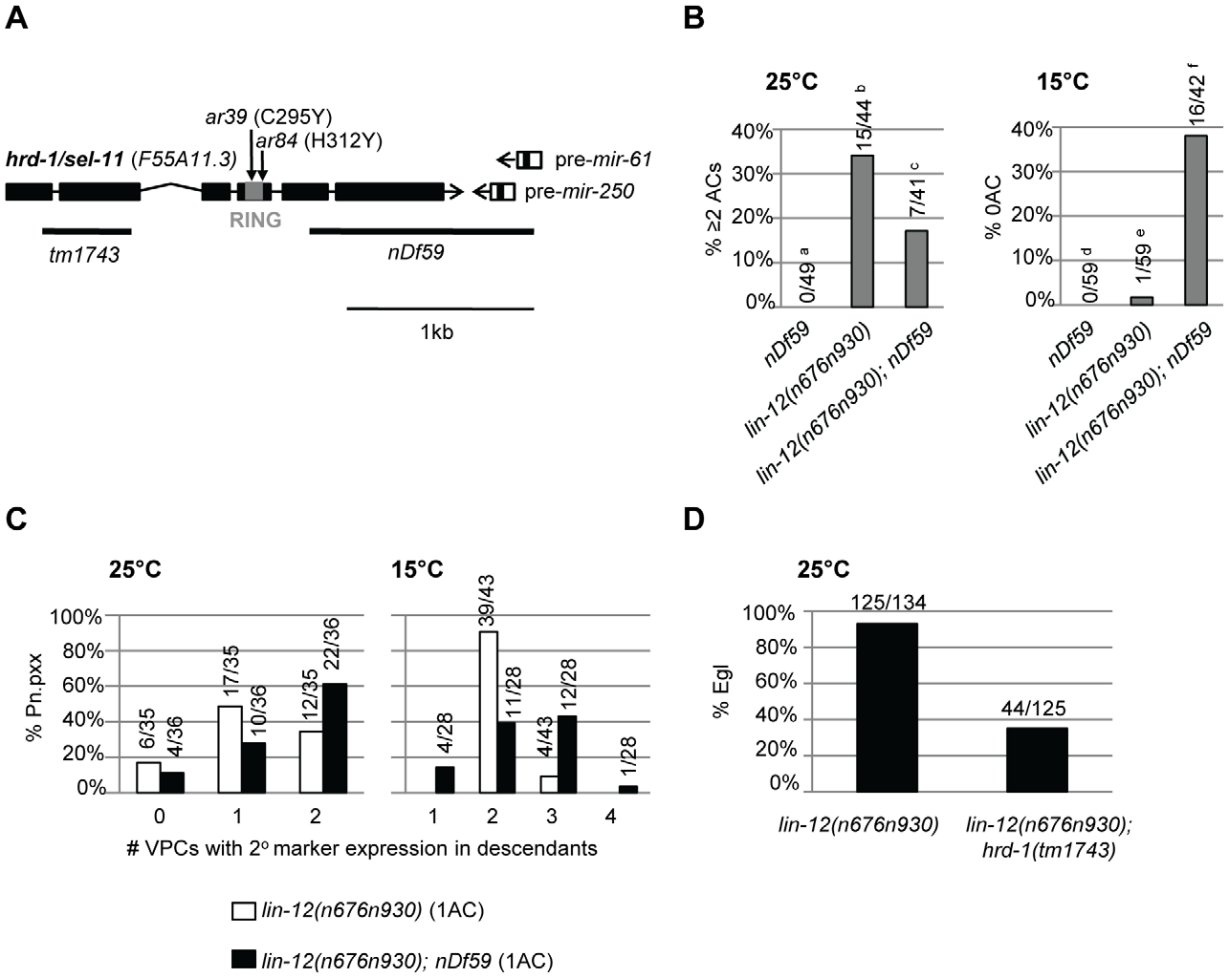
*nDf59* enhancement of *lin-12* activity and the identification of *sel-11* as *hrd-1*. (A) The *mir-61/250* genomic region. The coding regions for the pre-microRNAs of *mir-61* and *mir-250* are separated by only 44bp, making it likely that these two miRNAs are cotranscribed. Expression of *mir-61/250* in P5.p and P7.p depends on LIN-12 activation, mediated by LAG-1 Binding Sites in the 5′ flanking region [11]. The thick black lines indicate genomic regions deleted by *tm1743* or *nDf59* (see RESULTS). The RING domain of *hrd-1/sel-11* is indicated by a grey box. Molecular lesions were found in the RING domain of *hrd-1/sel-11* for two *sel-11* alleles, *ar39* and *ar84*, as indicated by arrows. These two alleles were found to contain point mutations in the RING finger domain of *hrd-1*, mutating either a histidine to a tyrosine (*ar84*, H312Y), or a cysteine to a tyrosine (*ar39*, C295Y), thereby mutating amino acids potentially important for the ubiquitination function of the RING domain. (B, C) *lin-12(n676n930)* has reduced activity at 25°C and mildly elevated activity at 15°C. The VU fate in the somatic gonad is determined by high *lin-12* activity, as is the 2° vulval cell fate. (B) *nDf59* decreases the number of anchor cells (AC) in the *lin-12(n676n930)* background at both 25°C and 15°C, which indicates an enhancement of *lin-12* activity. The number of ACs observed for each strain is as follows: ^a^ 49/49 1 AC. ^b^ 29/44 1 AC, 13/44 2 ACs, 2/44 3 ACs. ^c^ 34/41 1 AC, 7/41 2 ACs. ^d^ 59/59 1 AC. ^e^ 56/59 1 AC, 2/59 2 ACs. ^f^ 25/42 1AC, 1/42 2 ACs. Full genotypes of animals scored for AC marker expression are *unc-32(e189); arIs51[cdh-3::gfp]; nDf59* (GS5161), *unc-32(e189)lin-12(n676n930); arIs51* (GS4894), and *unc-32(e189)lin-12(n676n930); arIs51; nDf59* (GS5015). (C) *nDf59* increases the number of VPCs expressing a 2° marker in the *lin-12(n676n930)* background at both 25°C and 15°C. When scoring for effects in the VPCs, we scored only animals with 1 AC to avoid the influence of altered numbers of ACs on VPC fate in the *lin-12(n676n930)* background. In a wild-type background, P5.p and/or P7.p express the 2° fate marker *nIs106[lin-11::gfp]* (Table 1, [11]). At 25°C, some *lin-12(n676n930)* animals have less than two VPCs that express a 2° fate marker; this phenotype is suppressed by *nDf59*. At 15°C, some *lin-12(n676n930)* animals have more than two; this phenotype is enhanced by *nDf59*. Full genotypes of animals scored for expression of the *nIs106[lin-11::gfp]* 2° fate marker are *unc-32(e189)lin-12(n676n930); nIs106* (GS5014), and *unc-32(e189)lin-12(n676n930); nDf59; nIs106* (GS5077). (D) *hrd-1(tm1743)*, a null allele, suppresses the egg-laying defect (Egl) of *lin-12(n676n930)* at 25°C. Egl: egg-laying defective.

The possibility that *nDf59* might affect a negative regulator of *lin-12* activity arose when we examined the genetic interactions between *nDf59* and *lin-12(n676n930)*, a hypomorphic allele of *lin-12* in which activity is compromised but not eliminated [10]. When *lin-12(n676n930)* hermaphrodites are grown at 25°C, they exhibit defects caused by reduced *lin-12* activity: a highly penetrant egg-laying defect; a partially penetrant 2 AC defect; and, in hermaphrodites with 1 AC, a partially penetrant lateral signaling defect (P5.p and/or P7.p lose expression of a 2° fate marker) ([10]; Fig. 2B and 2C). At 15°C, *lin-12(n676n930)* retains mildly elevated *lin-12* activity due to the presence of the *n676* mutation. According to the proposed circuit, loss of *mir-61* should decrease *lin-12* activity; thus, we would expect that *nDf59* might enhance phenotypes caused by *lin-12(n676n930)* at 25°C and suppress phenotypes of *lin-12(n676n930)* at 15°C. However, we obtained the opposite results, that *nDf59* suppressed the loss-of-function phenotypes of *lin-12(n676n930)* at 25°C, and furthermore, enhanced the weak hypermorphic phenotype of this allele at 15°C (Fig. 2B and 2C). These results indicate that *nDf59* increases *lin-12* activity.

*nDf59* had been reported to remove 1143 bases encompassing *mir-61* and another microRNA, *mir-250* [12]. We independently sequenced the *nDf59* allele and confirmed the deletion breakpoints reported in WormBase (www.wormbase.org). During this process, we realized that, in addition to removing *mir-61/250*, *nDf59* also removes part of the adjacent protein-coding gene, *hrd-1* (Fig. 2A) (see MATERIALS AND METHODS). *C. elegans hrd-1* [13] is the ortholog of yeast *HRD1*, which was identified in a genetic screen for genes that mediate HMG-CoA reductase degradation in *Saccharomyces cerevisiae* [14]. Another gene defined in the same screen, *HRD3*, corresponds to the *C.* elegans gene *sel-1*, the first molecularly characterized negative regulator of *lin-12* [8], [15], [16].

In view of the functional relationship between *HRD1* and *HRD3* in yeast, we wondered if the genetic enhancement of *lin-12(n676n930)* by *nDf59* may be explained if *hrd-1*, like *HRD3*/*sel-1*, is a negative regulator of *lin-12* activity. This inference was confirmed by showing that *hrd-1(tm1743)*, an apparent molecular null allele, suppresses the egg-laying defect (Egl) of *lin-12(n676n930)* at 25°C (Fig. 2D).

The map position of *hrd-1* suggested that it might correspond to *sel-11*, previously defined as a negative regulator of *lin-12* [8]. We therefore sequenced the two available alleles of *sel-11,* and found that they contain point mutations in the RING finger domain of HRD-1 (Fig. 2A). *hrd-1* has been renamed *sel-11*, the first published name for the gene, in accordance with the accepted nomenclature convention in the field. The finding that two point mutations in the RING finger domain behave like a deletion of the gene suggests that the ubiquitin ligase activity of SEL-11 is important for negative regulation of *lin-12* activity.

### Genetic interactions implicate *cdc-42* as a negative regulator of *lin-12/Notch* activity

As mentioned above, *nDf59* deletes the microRNA *mir-250*, which appears likely to be cotranscribed with *mir-61* in response to LIN-12 activation. Using the microRNA Registry release 2.0 prediction of the *mir-250* sequence [17], we identified potential target genes computationally, using the same criteria that we used for *mir-61* targets: we required that 3′ UTRs have at least 7 bases of perfect complementarity to the 5′ end of *mir-250*, with this putative “seed match” conserved in the *C. briggsae* orthologs of the *C. elegans* genes [11]. Our interest in *cdc-42*, a candidate identified in this way, was stimulated when we found that *cdc-42(RNAi)* enhanced *lin-12* activity in sensitized backgrounds.

**Figure 3:**
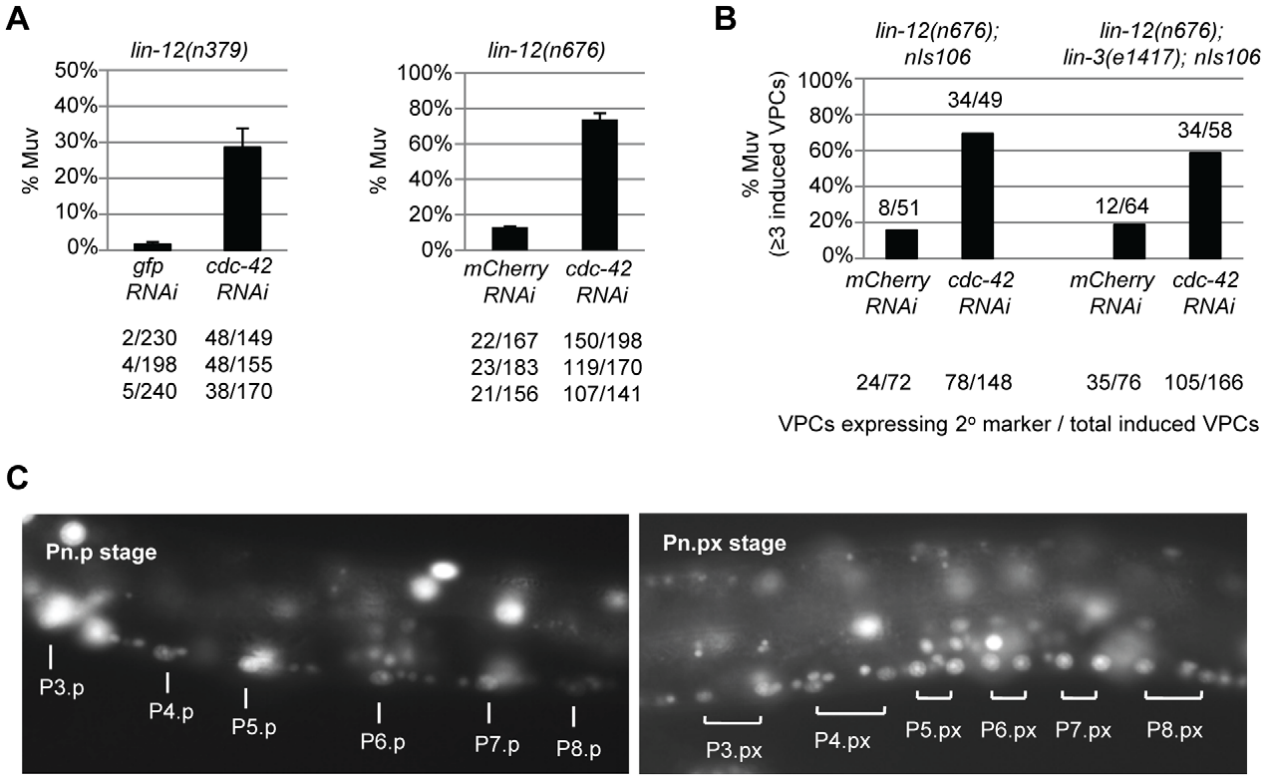
*cdc-42*(RNAi) enhances *lin-12* activity in the VPCs. (A) *cdc-42(RNAi)* enhances the Multivulva (Muv) phenotype of weak *lin-12(d)* alleles. Upon RNAi treatment, *cdc-42* enhanced the Muv phenotype of *lin-12(n379)*, as well as *lin-12(n676)*. Muv is defined as three or more ventral protrusions. (B) The Multivulva phenotype of *cdc-42(RNAi); lin-12(n676)* hermaphrodites is not altered by *lin-3(e1417)*, a mutation that reduces inductive signal expression. Animals were scored at the L3 Pn.pxx stage, where the VPCs have divided twice. The numbers of VPCs expressing the 2° fate marker *nIs106[lin-11::yfp]* are shown below. (C) A *cdc-42* transcriptional reporter is expressed in the VPCs and their descendants. The transcriptional reporter was generated by fusing the 5′upstream region of *cdc-42* to a *2nls-yfp* reporter with an *unc-54* 3′UTR. Expression of the reporter was strong and uniform in all six VPCs at the Pn.p stage and was upregulated in the daughters of P5.p, P6.p and P7.p.

The backgrounds we used were afforded by the mild hypermorphs *lin-12(n379)* and *lin-12(n676)* (Fig. 3A). These mild hypermorphs lack an anchor cell and their VPCs generally behave as wild-type VPCs do in the absence of the inductive signal, i.e. they all adopt the 3° fate (Fig. 1). If *lin-12(n379)* or *lin-12(n676)* activity is increased, some or all of the VPCs adopt the 2° fate, resulting in a “Multivulva” phenotype that is visible in the dissecting microscope [8], [18], [19] (Fig. 3A). We verified that the Multivulva phenotype of *cdc-42(RNAi); lin-12(n676)* reflects an increased number of VPCs adopting the 2° fate using a transgenic marker (Fig. 3B). These observations are consistent with a role of *cdc-42* as a negative regulator of *lin-12* activity in the VPCs.

We note that in a *lin-12(+)* background, *cdc-42(RNAi)* had been shown to cause a supernumerary anchor cell, which induces an extra VPC to adopt the 1° fate [9]. However, the Multivulva phenotype of *cdc-42(RNAi); lin-12(d)* hermaphrodites appears to reflect enhanced *lin-12(d)* activity in the VPCs rather than supernumerary anchor cells or increased production of inductive signal. First, *cdc-42(RNAi); lin-12(n676)* hermaphrodites, like control *lin-12(n676)* hermaphrodites, do not have any anchor cells: in three independent experiments, *cdc-42* RNAi performed on *lin-12(n676); arIs51* animals resulted in 0/51, 0/80, 1/63 L3 animals that had AC marker expression, compared to 1/51, 0/73, 0/55 for control *mCherry* RNAi-treated animals. Second, the Multivulva phenotype of *cdc-42(RNAi); lin-12(n676)* hermaphrodites is not altered by *lin-3(e1417)*, a mutation that reduces inductive signal expression (Fig. 3B). Third, as described above, in *cdc-42(RNAi); lin-12(n676)* hermaphrodites, extra VPCs adopt the 2° fate rather than the 1° fate.

We generated a transcriptional reporter containing the 5′ flanking region of *cdc-42*. This reporter drives strong, uniform expression in all six VPCs, and the level of expression appears to increase in the daughters of P5.p, P6.p and P7.p (Fig. 3C). The presence of *cdc-42* in VPCs and their descendants is consistent with the multiple roles of *cdc-42* in VPC development and specification inferred by Welchman [9] and the genetic interactions with *lin-12* described here.

These observations are consistent with a role of *cdc-42* as a negative regulator of *lin-12* activity in the VPCs. Phenotypes caused by the expression of dominant-negative versions of *Drosophila* ortholog of *cdc-42* have also suggested a role as a negative regulator of Notch signaling in wing development [20]. However, we were unable to obtain evidence that *mir-250* negatively regulates *cdc-42* in any assay (data not shown). When a revised *mir-250* sequence was published (miRBase 10.1, [21]), it was evident that *mir-250* has two additional adenines at its 5′ end that would preclude the appropriate seed match base pairing with *cdc-42*. Thus, although *cdc-42* behaves genetically as a negative regulator of *lin-12*, it is not likely to be regulated by *mir-250* in an analogous way to the LIN-12-mir-61-VAV-1 circuit.

## Discussion

We have described here two negative regulators of *lin-12/Notch* activity, *sel-11* and *cdc-42*. We discuss possible mechanisms by which these genes may influence *lin-12/Notch* activity in terms of our findings and relevant literature on their mammalian orthologs.

SEL-11/Hrd1p and a previously-described negative regulator, SEL-1/Hrd3p [15], [16], are both involved in the Hrd1p pathway of ER-associated degradation (ERAD). The Hrd1p pathway specifically targets proteins with misfolded lumenal domains for degradation. SEL-11/Hrd1p is the central E3 ubiquitin ligase, and SEL-1/Hrd3p acts in the recognition of terminally misfolded substrates [22], [23]. All *lin-12* alleles that display genetic interactions with *sel-1* and *sel-11* [8], [15], [16] carry mutations that alter the extracellular domain, which would be positioned in the ER lumen during protein synthesis and folding [24], [25]. Loss of the Hrd1p quality control system likely increases the stability or export of these mutant LIN-12 proteins.

There is some evidence that increased expression of SEL1L, the human SEL-1 ortholog, correlates with a decrease in tumor aggressiveness (reviewed in [4]), but if this correlation reflects effects on Notch activity is not clear. In addition, the human SEL-11 ortholog, Synoviolin, is essential for development in mice and has been implicated in hyperproliferation of synovial tissues in rheumatoid arthritis and in the degradation of p53 and IRE1 [26], [27], [28]; furthermore, there appears to be multiple modes of crosstalk between Notch and p53 [29]. These observations are consistent with the possibility that loss of *sel-11* might lead to elevated LIN-12/Notch activity in cancer.

*C. elegans* CDC-42 is a member of the Cdc42 subfamily of Rho family GTPases. Cdc42 has been implicated in many different cellular processes including polarity, cytoskeleton reorganization, vesicle trafficking, and signal transduction [30], [31]. The VPCs are polarized epithelial cells, with LIN-12 localized to the apical domain [32] and LET-23/EGFR localized to the basolateral domain [33]. Reciprocal negative regulation of endocytosis and trafficking of these receptors helps ensure proper pattern formation [11], [34], [35], [36], [37], [38], [39]. CDC-42 may contribute to this regulation through effects on VPC polarization and apical membrane organization, or through a general effect on endocytic traffic [40], [41], [42]. CDC-42 may affect LIN-12 signaling per se or through promoting the activity of the LET-23/EGFR-Ras-MAPK pathway: although genetic enhancement of *lin-12(d)* mutations was observed in the absence of the anchor cell, the normal source of inductive signal, it is possible that VAV-1 and CDC-42 affect a basal activity of LET-23 or another receptor tyrosine kinase that promotes Ras activity.

Cancer results from aberrations in cell-cell interactions and growth control, processes that are influenced by Notch activity. Depending on the cellular context, Notch can function as either a tumor suppressor (promoting differentiation) or proto-oncogene (promoting proliferation and/or suppressing apoptosis) [3]. In these roles, Notch crosstalks with p53 and Rho/CDC42 effectors [29], [43]. Our results raise the possibility that Cdc42 has effects on tumorigenesis through effects on Notch activity.

As mentioned in RESULTS, all of the *lin-12* alleles used in this study carry missense mutations that alter the extracellular domain and cause constitutive LIN-12 activity; in some cases, the *lin-12* alleles are composites of such activating mutations combined with second-site revertants that lower *lin-12* activity. The missense activating alleles alter a negative regulatory domain [24] now known to be revealed under normal conditions by ligand binding (reviewed in [44]). Equivalent missense mutations in human Notch1 cause T-ALL and perhaps other cancers [45]. Whether SEL-11/Hrd1p/Synoviolin and CDC-42/Cdc42p act to promote the activity of wild-type LIN-12/Notch or only these missense mutant forms is not clear. Nevertheless, the clear effect on the LIN-12/Notch missense mutant forms associated with cancer suggest that the reduction in the activity of these negative regulators may be associated with cancer formation or progression.

## Materials and Methods

### Strains and genetic analysis

*Caenorhabditis elegans* var. Bristol strain N2 was the wild-type parent strain of all mutants and markers used. Key strains used herein were: GS4944 *nDf59* (backcrossed to N2 4x), GS4420 *ayIs4 [egl-17::gfp]*, GS5043 *ayIs4; nDf59*, 

GS5775 *nIs106 [lin-11p::gfp],* GS5009 *nDf59; nIs106,* GS3804 *pha-1(e2123); arIs101 [K09H11.1p::yfp],* GS5010 *nDf59; arIs101,* GS5161 *unc-32(e189); arIs51[cdh-3::gfp]; nDf59,* GS4894 *unc-32(e189)lin-12(n676n930); arIs51,* GS5015 *unc-32(e189)lin-12(n676n930); arIs51; nDf59,* GS5014 *unc-32(e189)lin-12(n676n930); nIs106,* GS5077 *unc-32(e189)lin-12(n676n930); nDf59; nIs106,* GS3067 *unc-32(e189)lin-12(n676n930),* GS5186 *unc-32(e189)lin-12(n676n930); hrd-1(tm1743) V,* GS104 *unc-32(e189)lin-12(n676n930); sel-11(ar39),* GS257 *unc-32(e189)lin-12(n676n930); sel-11(ar84),* GS3196 *lin-12(n379),* GS23 *lin-12(n676),* GS4481 *lin-12(n676); nIs106 [lin-11p::gfp],* GS5098 *lin-12(n676); lin-3(e1417); nIs106.*

GE24 *pha-1(e2123)* was used as the recipient to create transgenes. All strains were grown using standard procedures at 20°C unless otherwise noted, except for strains with *pha-1(+)*-containing transgenes in the *pha-1(e2123)* background, which were maintained at 25°C. For strains that were scored at 25°C or 15°C, animals were maintained at the temperature of interest for at least two generations prior to scoring.

A strain carrying *hrd-1(tm1743)*, backcrossed 4 times, was the gift of S. Arur and T. Schedl.

### Transgenic lines

*cdc-42p::2nls-yfp::unc-54 3′UTR* reporter lines were made by injecting fusion PCR products [46] into *pha-1(e2123)* animals: *pha-1(e2123); arEx843-847* [*cdc-42p::2nls-yfp::unc-54 3′UTR* (0.2 ng/ul), pBX (50 ng/ul)].

### Fusion PCR products and plasmids

Fusion PCR was performed as reported [46]. ‘A’ primers and ‘B’ primers are forward and reverse primers, respectively, used to amplify promoter regions. ‘A*’ primers are nested forward primers used with the D* primer (see [46]) for the fusion step. The following primers were used to amplify the *cdc-42* 5′ region: AY56-A (CAATGGGCGATCAGGGTGTCTAT), AY56-A* (GCTAATAACCCGCACGGAGTAATG), AY56-B (agtcgacctgcaggcatgcaagctACTTGATCGTCTGCTTTTCGCCTG).

### RNAi experiments

Feeding RNAi experiments were performed at 20°C as described [47], [48]. Briefly, gravid adults were bleached and the eggs were placed on plates seeded with HT115 cells expressing the dsRNA of interest. T7 polymerase expression in the HT115 cells had been induced with 6 mM IPTG for at least four hours at room temperature before plating the eggs. To score the number of pseudovulvae at the adult stage, animals were scored three days after eggs were placed on plates. To score at the L3 Pn.pxx stage, animals were scored roughly 45 hours after eggs were plated.

### Imaging

All microscopy done on live animals was performed on a Zeiss Axioplan2 microscope, with a consistent exposure time used for each marker assayed.

### Sequencing the *hrd-1* genomic region of *sel-11* alleles

The full genomic region of *hrd-1*, gene-to-gene, was amplified by PCR in two fragments for sequencing, using the primer pairs hrd-1-F1 (ATTGATATGGCACATTCAGAGCTTG)/hrd-1-R1 (GTTGTTGTGGAAATCCAAACTGATG), and hrd-1-F2 (CATTGTCTCCGCAGTTGGTTCC)/hrd-1-R2 (CATCGTCCTCTTTTTGTTCTGCTG). Two different alleles of *sel-11*, *sel-11(ar39)* and *sel-11(ar84)*, were associated with two different alterations in the *hrd-1* gene (see RESULTS). We note also that strains lacking *sel-11* alleles but containing “*sel(arX)*”, a mutation that is in the background of some strains [8], did not contain any alterations in the *hrd-1* gene.

### Confirmation of deletion breakpoints for *nDf59* and *tm1743*

Genomic DNA was extracted from backcrossed *nDf59* and *tm1743* strains, and the full genomic region of *hrd-1* was amplified by PCR using the two primer pairs hrd-1-F1/hrd-1-R1, hrd-1-F2/hrd-1-R2 noted in the previous section. The deletion breakpoints for *nDf59* and *tm1743* reported in WormBase (www.wormbase.org) were verified by sequencing. In summary, *nDf59* removes 1143 bp, and the deletion breakpoints for *nDf59* are TGGATTTCCACAACAACCAGCTGGTGCC/GGAGGTGCTCAGCCTGG…GTTCTAGTCATTGCC/ATACGGAGGAAGGACTAAGC. *tm1743* is a 473 bp deletion with a 38 bp insertion: the deletion breakpoints are TCATGTTCCAATTGCTCAAGTCT/ATTTTATTCGGAGATTTGAGAG… CTTAGGAATCCTCAATCTTGGGA/TAACAAAGCCGTTTACCTGCTCT, and CAAAGTTTCTTGTATATCTCATGTTCCAATTGCTCAAG is inserted. The published information about *nDf59* only notes that *mir-61/250* is deleted [12], but F55A11.3/*hrd-1* is also affected (see WormBase and Fig. 2A).
